# Multimodal imaging of retinal pigment epithelial detachments in patients with C3 glomerulopathy: case report and review of the literature

**DOI:** 10.1186/s12886-017-0602-4

**Published:** 2017-11-22

**Authors:** Valeria Kheir, Ali Dirani, Matthieu Halfon, Jean-Pierre Venetz, Georges Halabi, Yan Guex-Crosier

**Affiliations:** 1 Jules Gonin Eye Hospital, Department of Ophthalmology, University of Lausanne, Ave de France 15, 1000 Lausanne 2, Switzerland; 2 Service de Néphrologie du CHUV, Université de Lausanne, Lausanne, Switzerland; 3 Centre hospitalier universitaire vaudois (CHUV), Centre de transplantation d’organes, Université de Lausanne, Lausanne, Switzerland

**Keywords:** Complement 3 glomerulopathy, Drusenoid pigment epithelial detachments, Alternative pathway, Multimodal imaging, Optic coherence tomography angiography, Choroidal neovascularization

## Abstract

**Background:**

To describe the optical coherence tomography angiograhy (OCTA) of drusenoid pigment epithelial detachments (PEDs) in a woman affected by Complement 3 (C3) glomerulopathy, which represents a spectrum of glomerular diseases characterized on fluorescent microscopy by *C3* accumulation with absent, or scanty, immunoglobulin deposits. It is due to acquired or genetically defective alternative pathway control and is generally associated with drusen-like deposits in Bruch’s membrane, as well as choriocapillaris. These retinal lesions can be associated with choroidal neovascularization and central serous chorioretinopathy (CSCR). OCTA is useful to detect neovascularization without injecting a contrast product, particularly in these patients who may have renal insufficiency.

**Case presentation:**

A 28-year-old woman affected by C3 glomerulpathy was diagnosed with asymptomatic multiple bilateral PEDs during a routine ophthalmologic consultation. To better characterize the lesions, multimodal imaging was performed and included: optic coherence tomography (OCT), en-face OCT, OCTA, fluorescence and indocyanine angiography. The OCTA clearly identified vascular network rarefaction with decreased choriocapillary vascularization. It confirmed that PEDs associated with C3 glomerulonephritis are not vascularized, but rather of serous type.

**Conclusions:**

Patients affected by C3 glomerulopathy can develop neovascular membranes as retinal complications of pigment epithelial detachments. Optical coherence angiography may be indicated to identify this complication, without injecting any contrast product that could produce further kidney damage.

## Background

Complement 3 (C3) glomerulopathy is a rare group of glomerular diseases. Presenting features include proteinuria, sometimes with nephrotic syndrome, hematuria, hypertension and renal failure. It mostly affects older children, adolescents and young adults. It is characterized by C3 deposits in the mesangium and along the glomerular basement membrane (GBM). In some patients, C3 glomerulopathy can be associated with retinal lesions described as drusen-like deposits in Bruch’s membrane and choriocapillaris. Thickening of GBM along with glomerular proliferative lesions induce impairment of glomerular filtration, hematuria, proteinuria and at end stage a loss of renal function. In the eye, thickening of Bruch’s membrane and subendothelial/subepithelial deposits induce the formation of drusen, predisposing to a risk of decreased vision [1, 2].

We present a patient with C3 glomerulopathy associated with retinal lesions, and report multimodal imaging findings. We also report a review of the literature of published cases of patients with C3 glomerulopathy associated with retinal findings, with a special focus on imaging studies.

## Case presentation

**Fig. 1:**
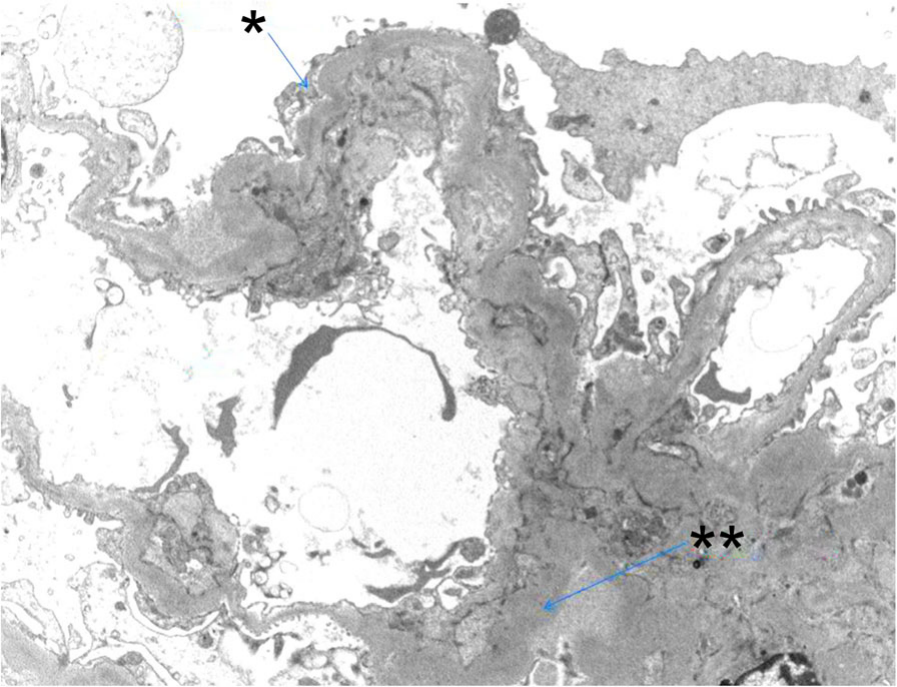
Electronic microscopy of a glomerulus shows the deposits localized in a subendothelial manner, as typically seen in C3 Glomerulonephritis C3GN (former MPGN type 1). * Podocyte foot process and ** Subendothelial deposit. Image kindly provided by Samuel Rotman MD pathology department CHUV)

A 28-year-old female, known to have C3 glomerulopathy, presented to our clinic for a routine check-up and was found to have asymptomatic multiple bilateral retinal pigment epithelium detachments (PEDs). At twelve years of age, the patient suffered a nephrotic syndrome,and a first renal biopsy revealed diffuse membranoproliferative glomerulonephritis (MPGN). This was treated with oral corticosteroids and cyclophosphamide and was maintained on azathioprine. Relapse occurred after a five-year period of remission, and a new renal biopsy showed a membranoproliferative glomerulonephritis of type I (MPGNI). Immunofluorescence assay was positive for deposits of C3 complement, and the diagnosis of C3 glomerulonephritis (C3GN) was confirmed. The electronic microscopy of a glomerulus showed that the deposits were localized in a subendothelial manner, as typically seen in C3 Glomerulonephritis C3GN (former MPGN type 1). (Figure 1). Secondary causes of C3GN were excluded by a complete workup: hepatitis B and C serology, cryoglobulinemia, absence of monoclonal gammapathy on serum electrophoresis. Complementary exploration in the serum confirmed an activation of the alternative pathway: C3 low at 0.23 g/l (n: 0.75–1.4), C4 normal at 0.26 g/l (n: 0.10–0.34), C5B9 elevated at 551.4 ng/ml (n: 127–303) and CH50 at 23% (n: 70–140%). Genetic and acquired autoantibodies (C3 nephritic factor, anti-factor H antibodies) investigations were negative. Treatment with low-dose cyclosporine resulted in stabilization of the disease for a further 5 years. At this point multiple relapses occurred with recurrence of proteinuria and decrease in renal function, treated with cyclosporine, followed by intravenous IV Rituximab. Despite the use of immunosuppressant drugs, proteinuria progressively worsened and renal function deteriorated. Peritoneal dialysis was started 1 year ago and the patient is currently evaluated for a renal graft. She is also on acenocoumarol for a factor V Leiden.

**Fig. 2:**
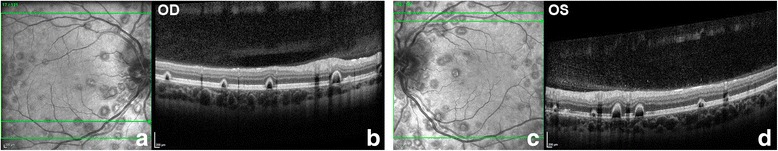
**a**. RE IR photography. **b**. RE OCT section performed through drusen. **c**. LE IR photography. **d**. LE OCT section performed through drusen. Multiple PEDs with serous content are visible on OCT

**Fig. 3:**
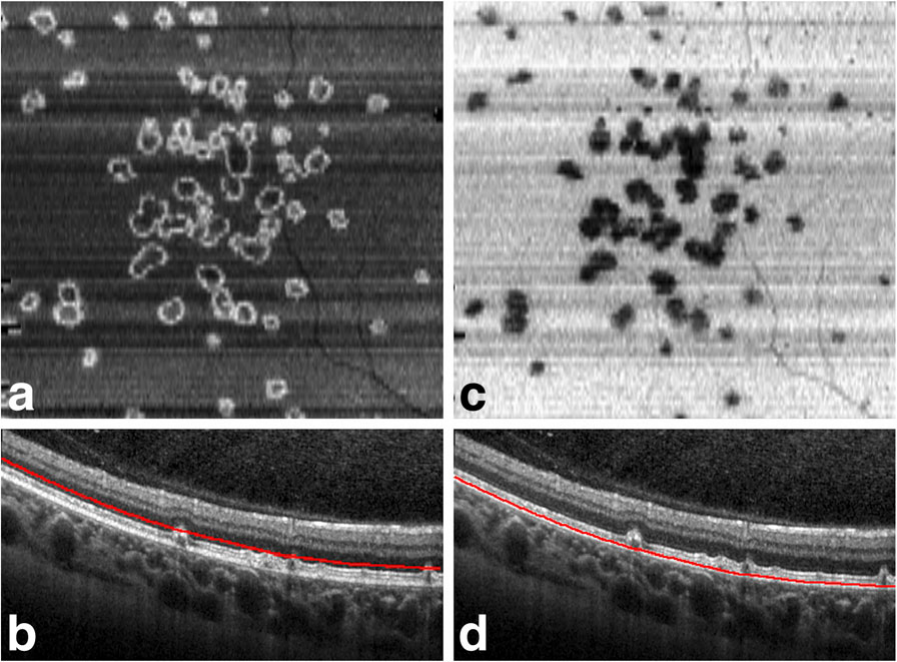
**a** and (**b**): RE Enface OCT at the external limiting membrane level. **c** and (**d**): RE Enface OCT at the RPE level. The deposits extend between the subepithelial space and external limiting membrane

**Fig. 4:**
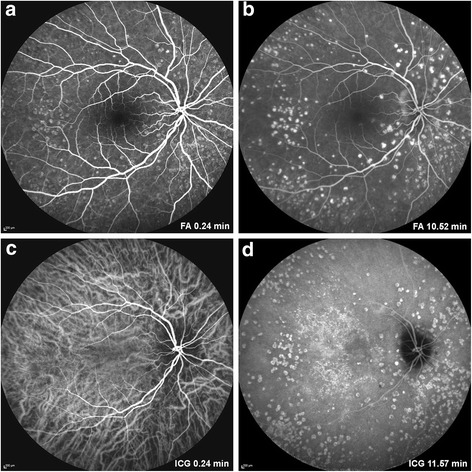
**a** and **b**: RE fluorescein angiography shows hyperfluorescent well circumscribed lesions on early and late time frames. **c** and **d**: RE indocyanine angiography at early and late phase: late hypercyanescence of the retinal lesions in both eyes

**Fig. 5:**
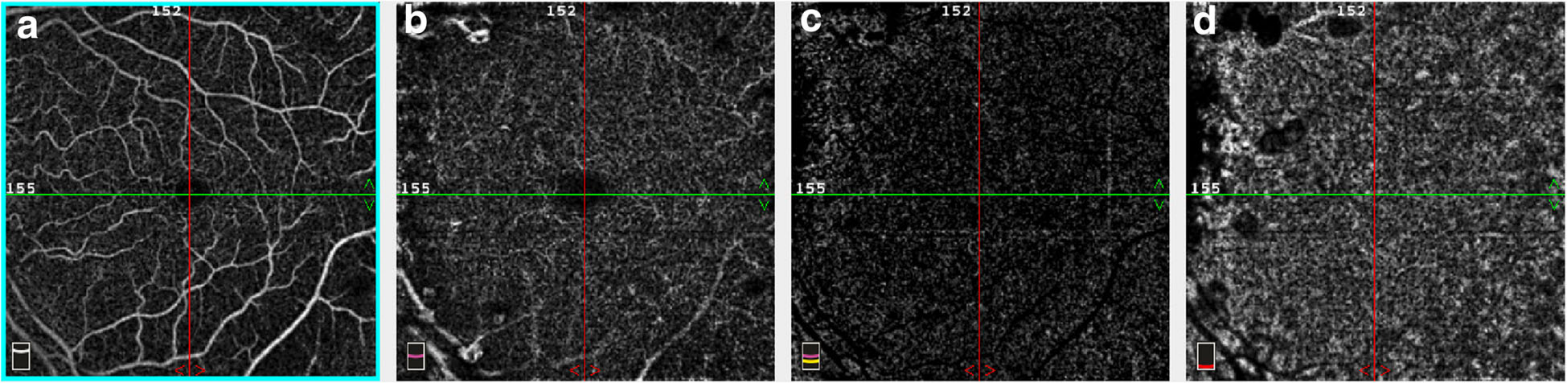
LE OCT angiography (OCTA). **a** and (**b**): In the superficial and deep capillary plexus: no abnormalities. **c** and (**d**): In the outer retina and choriocapillaris layers: no vascular membranes associated with PEDs, but rarefaction of vascular network, with a decrease of blood vessels in the choriocapillaris layer

At presentation, the patient had a routine ophthalmologic examination: best corrected visual acuity was 12/10 in both eyes, bilateral anterior segment examination was unremarkable, eye pressure was normal in both eyes. Fundus examination revealed more than 50 yellowish subepithelial drusen-like elevations in macula, mid-periphery and periphery of both eyes. Spectral domain optical coherence tomography (SD OCT) showed that these lesions were PEDs with serous content (Fig. 2). Choroidal thickness evaluated by enhanced depth OCT was 328 μm in the right eye and 271 μm in the left eye. En-face OCT showed PED at different regions of the retina and clearly localized the deposits between the subepithelial space and the external limiting membrane (Fig. 3). Fluorescence angiography (FA) revealed hyperfluorescent well circumscribed lesions in a greater number than those observed in the fundus on early and late time frames, with no evidence of leakage. Indocyanine angiography showed late hypercyanescence of the retinal lesions in both eyes (Fig. 4). Optical coherence angiography (OCTA) of the superficial and deep capillary plexuses revealed no abnormalities. OCTA of the outer retina and choriocapillaris layers did not show any abnormal vascular membranes associated with PEDs, but vascular network rarefaction was clearly identified with fewer blood vessels in the choriocapillaris layer (Fig. 5). OCT angiography (OCTA) confirmed that PEDs associated with C3 glomerulonephritis were not vascularized but rather of serous type. Vascular abnormalities seen in the choriocapillaris layer may play a role in the pathogenesis, or simply represent a consequence of material accumulation in PED associated with C3 glomerulonephritis.

**Fig. 6:**
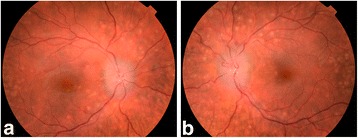
**a**. RE fundus. **b**. LE fundus. Bilateral papillary edema and bilateral multiple PEDs

The patient was followed regularly in our department. At her last follow up visit (2 years after presentation), she presented asymptomatic bilateral papillary edema (Fig. 6). Visual acuity was still 12/10 in both eyes, color vision (Ishihara pseudoisochromatic chart) was normal in both eyes, visual field was normal in right eye but showed small superior defects in the left eye. Anterior biomicroscopy was normal and bilateral PEDs remained present. Retinal nerve fiber layer thickness in the peripapillary area was increased: 248 μm in the right eye, and 264 μm in the left eye. Cerebral magnetic resonance imaging (MRI) was performed and revealed primary intracranial hypertension, for which she was referred to the neurosurgeons.

## Discussion and conclusions

In the past, classification of membranoproliferative glomerulopathy (MPGN) was based on pathological findings of electron microscopy, and 3 types of MPGN were described: type I is characterized by subendothelial deposits, type II or dense deposit disease (DDD) by glomerular basement membrane dense deposits, and type III by both subepithelial and subendothelial deposits [3]. Recently, a new classification has been proposed based on immunofluorescence microscopy: immune complex-mediated glomerulonephritis and complement-mediated glomerulonephritis [1, 4]. The new classification is more appropriate as it better reflects the underlying pathophysiology of the disease.

MPGN types I and III have been reclassified in complement 3 glomerulonephritis (C3GN), characterized by discrete deposits in the mesangium, and subendothelial and subepithelial capillary walls. MPGN II and C3GN are complement-mediated glomerular disease and are part of C3 glomerulopathies, these characterized by complement 3 deposits with absent or scant immunoglobulin deposits on fluorescent microscopy. C3 glomerulopathies represent a spectrum of glomerular diseases due to defective alternative pathway control [1, 2]. The alternative pathway recognizes and eliminates microbes or modified cells. It is now recognized that not only MPGN II, but also C3GN, can be accompanied by retinal drusen.

The choriocapillaris-Bruch membrane-retinal pigment epithelium (RPE) interface is anatomically similar to the capillary tuft-GBM-glomerular epithelial interface [5]. These organs lack local membrane-bound complement regulators, thus they are vulnerable to injury caused by products of uncontrolled alternative pathway. Deposits in retina and kidney have the same structure and composition [6].

Drusen are defined as deposits within Bruch’s membrane and are a hallmark feature of age-related macular degeneration (AMD). These lesions have been largely described in MPGN type II since 1989 [5–20], but the first description of drusen in MPGN type I was given in 2009 [21]. In a case report, Han et al. reported the presence of multiple “drusens” and “PEDs”. In 2001, Mullins et al. analyzed the structure and composition of drusen in MPGN and in post-streptococcal GN, and compared them with drusen in AMD. They found that the drusen have a similar composition in both types of membranous GN and in AMD: vitronectin (inhibitor of complement), complement C5, complement C5b-9, TIMP-3, amyloid P, and lipids. It was not specified whether MPGN was of type I or type II, but material within the drusen was electron-dense and electron-lucent [22]. The lesions observed in our patient were completely transluscent on OCT, representing serous PEDs. In the literature, various lesions such as drusen, PEDs, drusenoid PEDs were reported in patients with C3GN [21, 22]. These lesions may represent a continuum of the same phatophysiologic process involving the accumulation of material in the subretinal space. This may result in a decrease of retinal function, as revealed by abnormally lower elecro-retinography responses and reduced sensitivity in areas of high density.

MPGN II can be associated with choroidal neovascularization and central serous chorioretinopathy (CSCR)-like findings [9, 23–27]. As MPGN I and II are part of a spectrum of the same C3 glomerulopathies, patients with either type of disease should be regularly checked in order to identify and prevent ophthalmologic complications.

Various retinal imaging studies have been described in patients affected by C3 glomerulopathy. First of all, SD OCT is useful on a routine basis for all patients with retinal disease. It helps to define exactly which layers of the retina are affected by the disease, and allows the diagnosis of any secondary complication (choroidal neovascularization, CNV or atrophy). In the literature, many structural alterations in outer retinal layers and RPE were reported in patients with C3 glomerulopathy: irregularities in RPE surface, lucent or tent-shaped RPE detachments or elevations, sub-RPE drusenoid deposits, areas of compression of the photoreceptor layer, missing inner segments/outer segments IS/OS and external limiting membrane ELM back-reflection [1, 2, 11, 26, 28]. A prototype high-speed ultrahigh optic coherence tomography (UHR-OCT) with an axial resolution of approximately 3 μm in tissue was realized in the left eye of a 29-year-old man, affected by drusen-like deposits in the context of C3 glomerulopathy. The UHR-OCT showed not only RPE detachment, but also an irregular and prominent Bruch’s membrane measuring between 9 and 13 μm at 3 locations, compared to that of healthy patients being between 2 and 4.7 μm. It seems to be an interesting tool, because it permits the delineation of Bruch’s membrane from RPE, which is not possible with the commercially available SD-OCT that has an axial resolution in tissue of 5 to 7 μm [9].

Another useful exam is fluorescein and indocyanine green angiography. Drusen without any complications have been described as having a starry sky appearance on fluorescein angiography [2]. Early phase typically shows window defects due to basal lamina drusen, and late phases typically show staining of hyperfluorescence with no evidence of fluid leakage [1, 7, 13, 21, 29, 30]. Indocyanine green angiography helps in excluding or identifying choroidal neovascular membranes or CSCR-like lesions as possible complications [9, 21]. Fluorescein angiograms may show normal results, while penetrating ratios detected by vitreous fluorophotometry readings can be abnormally high, as demonstrated by Raines et al. in 1989. This indicates a breakdown of the blood retinal barrier as a consequence of retinal pigment epithelium dysfunction due to Bruch’s membrane deposits [17]. This imaging technique is not, however, used on a routine basis.

Other authors reported microperimetry results, and showed that eyes of patients with C3 glomerulopathy may have reduced sensitivity in areas of high drusen density, probably indicating areas of reduced retinal function [13, 28].

Electophysiologic tests can also provide information about retinal function. Leys et al. reported electro-oculogram (EOG) results to be normal in patients without choroidal neovascularization and abnormal in patients with choroidal neovascularization (reduced light peak/dark peak) on EOG [20]. Contrary to this, C O’Brien et al. reported 3 asymptomatic patients with typical drusen-like lesions seen in the posterior pole, who had abnormally low Arden ratios on EOG but normal elecro-retinography responses (PERG, Flash-ERG, Flicker ERG). This was the first report of choriocapillaris and Bruch’s membrane disease causing electroculographic abnormality without any visual deficit [31]. Lahbil et al. also reported a case of bilateral retinal drusen in a context of C3 glomerulopathy where the ERG was normal [30]. In the presence of retinal complications such as widespread chorioretinal atrophy, EOG, ERG, multifocal ERG and dark adaptation can all show abnormalities [2, 32].

Fluorescein angiography is a useful diagnostic test, but requires injection of fluorescein dye that can be contraindicated in patients with advanced kidney disease. Table 1 resumes the exams used by previous authors to describe drusen. We highlight in this paper the possibility of using OCT angiography in these patients. This is a new retinal imaging technique that allows analysis of the retinal plexuses and choroidal vessels without injecting any dye, and can also identify neovascular membranes. To our knowledge this is the first report of OCTA of multifocal PEDs associated with C3 glomerulopathy.

**Table 1:** This table resumes the exams used by previous authors to describe retinal drusen

Authors, Journal (Year of publication)	Number of cases	Symptoms	Clinical findings	Imaging Findings	Lesions
Savige J et al., Ophthalmic Genet (2016)	6	visual acuity normal or near normal initially. First symptom: impaired night vision, progression to loss of peripheral vision	bilateral symmetrical drusen. 2 types: 1. basal laminar drusen = small, numerous, yellow. 2. large soft whitish-yellow. Retinal atrophy after 15 years and choroidal neovascularization	OCT: irregularities in RPE surface, RPE detachments, neovascular membranes. Fluorescein angiography: starry sky appearance of druse, multiple small hyperfluorescent spots throughout retina and complications. Amsler grid:distorsion with late complications multifocal ERG: lower amplitudes and lower peak amplitude with retinal atrophy	typical drusen, PED, CNV and atrophy
Dalvin LA et al., Retin Cases Brief Rep (2016)	2	1. VA 20/300 in both eyes with eccentric fixation2. VA 20/20 inboth eyes	peripheral drusen, subretinal and RPE fibrosis, RPE hypertrophy, scarring	OCT: drusen Fluorescein angiography: areas of hypofluorescence corresponding to subretinal fibrosis surrounded by leakage, window defects, andmultiple drusen	typical drusen, subretinal fibrosis
Adhi M et al., Ophthalmic Surg Lasers Imaging Retina (2014)	1	progressive loss of vision secondary to CNV in RE, and new distorsion of vision in the LE	hemorrhage and subretinal fluid superonasal to the macula	OCT: RPE detachment, irregular and prominent Bruch’s membrane ICG angiography: no definitive signs of CNV	PED, suspicion of CNV
Empeslidis T et al., Case Rep Ophthalmol Med (2012)	1	problems with near vision tasks	signs consistent with RPE detachments and small drusen-like lesions	OCT: PEDs, intraretinal fluid in a cystoid form of less than 50um in the inner retinal layers. No subretinal fluidFluorescein angiography: early phase: window defect due to basal lamina drusen; late phase: staining of hyperfluorescence. No leakage. RPE layer intact.	typical drusen. PED, intraretinal fluid with no subretinal fluid. No CNV
Ritter M et al., Br J Ophthalmol (2010)	3	VA normal or slightly reduced	drusen: temporal in early cases, and throughout the retina in advanced cases	OCT: RPE elevations, areas of compression of the photoreceptor layer missing of IS/OS and ELM backreflection Microperimetry: reduced sensitivity in areas of high drusen density	typical drusen
Han et al., Arch Ophthalmol (2009)	1	VA normal in LE, and 20/30 in RE	bilateral multifocal, 200-300um yellowish lesions at choroid and subretinal pigment epithelial level	OCT: lucent focal elevations of RPE Fluorescein and indocyanine Angiography: staining of lesions throughout the fundi, more numerous than those observed by ophthalmoscopy	typical drusen
Amer Awan M et al., Clin Exp Optom (2008)	1	blurring vision, micropsia, metamorphopsia	elevated area overlying the macula in both eyes. Multiple pale areas without any drusen. Peripheral retina normal in both eyes.	OCT: tent-shaped RPE detachment with overlying detachment of neurosensory retina and loss of foveolar contour Fluorescein Angiography: early multiple hyperfluorescence areas corresponding to these pale areas. Mid-phase leakage of dye in subretinal space	atypical idiopathic serous central chorioretinopathy with spontaneous resolution at 6 weeks
Shenoy R et al., Eur J Ophthalmol (2006)	1	asymptomatic	multiple drusen-like lesions	Microperimetry: areas of reduced retinal sensitivity in areas which were laden with drusen-like deposits, probably indicating areas of reduced retinal function OCT: drusen Fluorescein Angiography: multiple window defects in the posterior pole of both eyes corresponding to the drusen-like lesions	typical drusen
Colville D et al., Am J Kidney Dis (2003)	1	poor night vision, complications resulted in severe visual loss	occasional drusen, widespread chorioretinal atrophy, macular pigmentation	dark adaption test: uniphasic- > consistent with severe rod dysfunction ERG: delayed rod and delayed combined rod and cone responses EOG: reduced light peak/dark through (or Arden ratio) possibly reflecting an ocbstruction to the passage of metabolites from the choriocapillaris Fluorescein angiography: subfoveal choroidal neovascularization	drusen, CNV and atrophy
Lahbil D et al., J Fr Ophtalmol (2002)	1	important decrease of visual acuity in both eyes	diffuse punctiform yellow subretinal lesions and central serous detachment in both eyes	Fluorescein Angiography: after resorbtion of serous fluid, punctiform hyperfluorescent lesions still staining at late phase. ERG:normal	CRSC- like lesion
Mullins RF et al., Eye (2001)	2		numerous subretinal RPE deposits similar to drusen seen in AMD	histochemistry/ immunohistochemistry: same composition of drusen in AMD	typical drusen
Polk T et al., Arch ophthalmol (1997)	1	blurred vision in right eye, with visual acuity 20/20 in RE, and 20/200 in LE	fine drusen in both eyes. Neurosensory detachment in the nasal macula of RE, and disciform scar in LE	Fluorescein agiography: multiple discrete hyperfluorescent spots corresponding to drusen, and early and late staining at RPE level in the area of the detachment, with 4 pinpoints sites of late leakage. Minimal pooling of dye in the subretinal space.	neurosensory detachment with spontaneous resolution at 2weeks
C O’Brien et al., Br J Ophthalmol (1993)	4	asymptomatic	multiple, yellow, drusen-like lesions	EOG: abnormal low Arden ratios PERG, flash ERG, and flicker ERG: waveform amplitudes essentially normal for all 4 subjects	typical drusen
Leys A et al., Graefes Arch Clin Exp Ophthalmol (1991)	23	–	small-sized subretinal nodules simulating basal laminar drusen. 4 patients displayed marked retinopathy and3 of them exhibited subretinal neovascularization	EOG: Light Peak/Dark peak ratio reduced in 4 (36%) of the 11 patients with EOG recordings. EOG and visual field: grossly normal in patients who did not exhibit choroidal neovascularization.	Typical drusen, CNV
Leys A et al., Pediatr Nephrol (1991)	3	–	granular retinal changes	Fluorescein angiography: numerous basal laminar drusen	drusen
Leys A et al., Eur J Ophthalmol (1991)	4	decrease of vision	drusenoid lesions	Fluorescein angiography: neovascular membranes	CNV
Duvall-Young J et al., Br J ophthalmol (1989)	1	VA 6/24 in both eyes	normal fundus	Fluorescein angiography: numerous hyperfluorescent discrete foci at posterior pole, becoming more intensely fluorescent with time	typical drusen
Duvall-Young J et al., Br J ophthalmol (1989)	11	asymptomatic	few drusen	Fluorescein angiography: few hyperfluoresent spots corresponding to scattered drusen	typical drusen
Raines MF et al., Br J Ophthalmol (1989)	5	asymptomatic	drusen	Vitreous Fluorophotometry: Breakdown of the blood retinal barrier: vitreous fluorophotometry readings and penetration ratios abnormally high, indicating that the deposits in Bruch’s membrane cause retinal pigment epithelial dysfunction. Fluorescein angiography: normal.	typical drusen

PEDs in MPGN have been shown to spontaneously resolve [29], but generally the longer the renal disease is present, the greater their evolution, becoming more numerous with later atrophic changes [12, 15, 16]. Subretinal membranes, macular detachment and central serous retinopathy may occur. Some treatments, such as corticosteroids, immunosuppressants and plasmapheresis have been used in these patients to treat the renal disease, but they did not improve the retinal condition. Recently, a newer drug was used in C3 glomerulopathy [intravenous eculizumab (monoclonal antibody that inhibits C5 and prevents formation of the C5b-9 membrane attack complex)] and resulted in substantial improvement of proteinuria, but it had no effect on retinal accumulation of cascade degradation products.

In patients with C3 glomerulopathy, regular ophthalmologic examination should be carried out in order to identify retinal complications early in their development. For patients having undergone a renal graft, ophthalmologic follow-up should be continued as the disease has been reported to recur in these cases [33].

Multimodal imaging of retina with OCT and OCT angiography could be useful in patients with C3 glomerulopathy, to help to identify early retinal manifestations of the disease and enable diagnosis of possible complications at an earlier stage. These patients may develop neovascular membranes, so OCTA appears to be a useful exam to identify this complication without the need to use contrast products that could affect renal function. Considering the progression of retinopathy and potential retinal complications in C3 glomerulopathies, close ophthalmologic follow-up is recommended, even after a renal graft.

## Abbreviations

AMD: Age-related macular degeneration
C3: Complement 3
C3GN: Complement 3 glomerulonephropathy
CSCR: Central serous chorioretinopathy
ELM: External limiting membrane
EOG: Electrooculogram
ERG: Electroretinogram
IS/OS: Inner segment /outer segment
MPGN: Membranoproliferative glomerulonephritis
OCTA: Optic coherence angiography
PEDs: Pigment epithelial detachments
PERG: Pattern-Electroretinogram
RPE: Retinal pigment epithelium
UHR-OCT: Ultrahigh-resolution optical coherence tomography
